# Physical activity during pregnancy and its relationship with gestational weight gain

**DOI:** 10.1590/1518-8345.6488.3876

**Published:** 2023-03-27

**Authors:** Enrique Ramón-Arbués, José Manuel Granada-López, Blanca Martínez-Abadía, Emmanuel Echániz-Serrano, Lucía Sagarra-Romero, Isabel Antón-Solanas

**Affiliations:** 1 Universidad San Jorge, Facultad de Ciencias de la Salud, Villanueva de Gállego, Aragón, Espanha; 2 Grupo de investigación TRANSFERCULT (H27_20D), Zaragoza, Aragón, Espanha; 3 Universidad de Zaragoza, Facultad de Ciencias de la Salud, Zaragoza, Aragón, Espanha; 4 Ayuntamiento de Zaragoza, Servicio de Prevención y Salud Laboral, Zaragoza, Aragón, Espanha; 5 Grupo de investigación GAIAS (S59_20D), Zaragoza, Aragón, Espanha; 6 Grupo de investigación GENIAPA (GIIS094), Zaragoza, Aragón, Espanha; 7 Grupo de investigación Seguridad y Cuidados (GIISA021), Zaragoza, Aragón, Espanha

**Keywords:** Motor Activity, Pregnancy, Body Weight Changes, Longitudinal Studies, Exercise, Nurse Midwives, Actividad Motora, Embarazo, Cambios en el Peso Corporal, Estudios Longitudinales, Ejercicio Físico, Enfermeras Obstetrices, Atividade Motora, Gravidez, Alterações do Peso Corporal, Estudos Longitudinais, Exercício, Enfermeiras Obstétricas

## Abstract

**Objective:**

to describe the physical activity patterns of a cohort comprised by pregnant women from our environment and to explore its association with weight gain in each of the trimesters of pregnancy.

**Methods:**

a descriptive and longitudinal study conducted with a sample of 151 women. The International Physical Activity Questionnaire was used to assess physical activity during pregnancy based on volume, intensity and setting where it is performed. Different multiple linear regression models were performed to analyze the association between physical activity and gestational weight gain

**Results:**

physical activity decreased during pregnancy, both in terms of time and intensity. Pre-gestational Body Mass Index was the main factor associated with lower weight gain throughout pregnancy. The influence of physical activity on gestational weight gain was limited to the third trimester of pregnancy, where an inverse association was observed between both variables.

**Conclusion:**

the results of this study show an important reduction in physical activity during pregnancy and suggest that it exerts a limited influence on gestational weight gain

## Abstract (es)

**Objetivo:**

describir los patrones de actividad física de una cohorte de gestantes de nuestro medio y explorar su asociación con la ganancia de peso en cada uno de los trimestres del embarazo.

**Método:**

estudio descriptivo longitudinal sobre una muestra de 151 mujeres. Se utilizó el Cuestionario Internacional de Actividad Física para evaluar la actividad física durante el embarazo en función del volumen, intensidad y ámbito de realización. Se llevaron a cabo diferentes modelos de regresión lineal múltiple para analizar la asociación entre actividad física y ganancia de peso gestacional.

**Resultados:**

la actividad física disminuyó durante el embarazo, tanto en tiempo como en intensidad. El índice de masa corporal pre-gestacional fue el principal factor asociado con una menor ganancia de peso a lo largo de todo el embarazo. La influencia de la actividad física sobre la ganancia de peso gestacional se limitó al tercer trimestre del embarazo en el que se observó una asociación inversa entre ambas variables.

**Conclusión:**

los resultados de este estudio muestran un importante descenso de la actividad física en la época del embarazo y sugieren una influencia limitada de ésta sobre la ganancia de peso gestacional.

## Abstract (pt)

**Objetivo:**

descrever os padrões de atividade física de uma coorte de gestantes em nosso meio e explorar sua associação com o ganho de peso em cada um dos trimestres de gestação.

**Método:**

estudo descritivo longitudinal com uma amostra de 151 mulheres. O Questionário Internacional de Atividade Física foi utilizado para avaliar a atividade física durante a gestação de acordo com o volume, intensidade e escopo do desempenho. Diferentes modelos de regressão linear múltipla foram utilizados para analisar a associação entre atividade física e ganho de peso gestacional.

**Resultados:**

a atividade física diminuiu durante a gestação, tanto em tempo quanto em intensidade. O índice de massa corporal pré-gestacional foi o principal fator associado ao menor ganho de peso ao longo da gestação. A influência da atividade física no ganho de peso gestacional limitou-se ao terceiro trimestre de gestação, no qual foi observada associação inversa entre ambas as variáveis.

**Conclusão:**

os resultados deste estudo mostram uma diminuição significativa da atividade física no momento da gravidez e sugerem uma influência limitada desta no ganho de peso gestacional.

**Highlights**

(1) Intensity and duration of physical activity decrease throughout pregnancy

(2) Physical activity decreases in all its scopes, except for playful activities (which increase).

(3) The factor that most influences weight gain is pre-gestational BMI.

(4) Physical activity only exerts an influence on weight gain in the 3rd trimester of pregnancy

(5) Midwives should monitor and promote physical activity during pregnancy

## Introduction

Overweight and obesity are two factors associated with high morbidity and mortality rates ^(1)^ and their prevalence has increased exponentially in recent years, to the extent of becoming priority Public Health problems(2-3). During pregnancy, excessive Gestational Weight Gain (GWG) is a risk factor for mothers and newborns alike, being related to complications such as macrosomia ^(4- 5)^ and delivery dystocia ^(6- 7)^ or to different maternal pathologies such as gestational diabetes(8-9) or hypertensive disorders during pregnancy ^(10- 11)^ . This circumstance leads to the need to explore those factors that may be related to better GWG control. One of these factors is the Physical Activity (PA) performed throughout pregnancy. 

PA during pregnancy carries along minimal risks for the mother-fetus dyad ^(12)^ . To the contrary, previous experimental studies have shown that performing PA throughout pregnancy might carry with it various benefits for women without contraindications for its practice, such as improved mood or prevention of musculoskeletal pain, gestational diabetes and hypertensive disorders during pregnancy, among others ^(13- 14)^ . 

In relation to the influence that the PA performed during pregnancy may exert on GWG, knowledge is more limited and research is scarce, especially in Spain. From the experimental point of view, this relationship has been tested in several clinical trials, mostly observing lower weight gains in the intervention groups when compared to the controls ^(15- 16)^ . There are fewer observational studies on the topic and their results have proved to be less concluding, even with contradictory results ^(17- 18)^ . In addition, most of these studies analyze the association between PA and GWG at the end of pregnancy through a single assessment of PA, generally playful or leisure-related, without taking into account the evolution it might have presented throughout pregnancy. Likewise, the GWG assessment is limited to the final number of kilograms gained during pregnancy ^(19- 21)^ . Based on these deficits, the objective of this research was to describe the PA patterns (in terms of volume, intensity and setting where it is performed) of a cohort comprised by pregnant women from our environment and explore its association with weight gain in each of the trimesters of pregnancy. 

## Method

### Study design and locus

A descriptive, longitudinal and prospective study conducted based on a sample of pregnant women from Sector III of the Autonomous Community of Aragon (Spain).

### Population

The participants were recruited during the second half of 2020 and first half of 2021, in Obstetrics follow- up consultations corresponding to the first trimester of pregnancy (< 13 gestational weeks).

The following initial inclusion criteria were considered: legal minority, multiple pregnancy, and presence of insurmountable language barriers. During the follow-up period, the women excluded from the analysis were those that underwent miscarriages or were diagnosed with some severe pregnancy-related problem such as delayed fetal growth, possible premature birth, hypertensive disorders during pregnancy or placental pathology, among others.

In order to calculate the minimum sample size required, the expected proportion of pregnant women with low PA levels was calculated by means of a pilot test conducted with 40 subjects (3rd trimester) from our environment. Consequently, for a 95% confidence level and 5% precision, the minimum number of participants to be included in the analysis should be 142. Given the prospective nature of the study, and foreseeing follow- up losses, a total of 220 pregnant women were recruited. From this total of 220, 4 participants were excluded due to miscarriage, as well as 8 due to pregnancy-related pathologies and 57 due to impossibility to establish phone contacts.

Three contacts with the participants were made throughout the follow-up period. The first one was in person and the others via telephone calls. Recruitment of the participants took place in the first of these contacts, during the pregnancy control visits in the 1st trimester of their pregnancies (mean of 12.1 gestational weeks). In this same contact, the participants self-reported their pre-gestational weight and their height was measured by duly trained personnel. Subsequently, they answered a general questionnaire with sociodemographic data, health-related habits and medical history.

In the second (mean of 22.3 gestational weeks) and third mean of 34.1 gestational weeks) trimesters of pregnancy, the participants were contacted in order to collect data related to their work status, the PA performed, weight gain, and possible onset of excluding factors for the research.

### Variables and instruments used for collection

At recruitment, data related to maternal age (years old) were collected, as well as to height, parity, history of previous miscarriages (yes or no), area of residence (rural or urban), geographical origin (native or immigrants), highest schooling level attained (Elementary School, High School or University studies), work status (active or not) and smoking habit at the beginning of pregnancy (yes or no).

The PA performed by the participants was assessed by means of the long version of the International Physical Activity Questionnaire (IPAQ) validated questionnaire, following the processing criteria described for the tool. IPAQ is a questionnaire based on recall of the PA performed in the last 7 days that has been recurrently used in the pregnant population ^(22- 23)^ and validated with the Spanish population ^(24)^ . It provides qualitative and quantitative information about the PA performed. From the qualitative point of view, it classifies the population into 3 PA levels (low, average or high). In addition, it quantifies PA by the settings in which it is performed (total, at work, for transportation, at home and playful). In the case of the PA quantitative analysis, the unit of measurement is Metabolic Equivalent of Task (MET), where 1 MET is the energy expenditure derived from the metabolic level at rest. 

The “pre-gestational weight” variable and in each of the trimesters of pregnancy was based on the participants’ self-report. With the pre-gestational weight and height, the participants’ pre-gestational Body Mass Index (BMI) was calculated, classifying them into the following groups: “low weight” (< 18.5 kg/m ^2^), “normal weight” (from 18.5 to 24.9 kg/m ^2^), “overweight” (from 25 to 29.9 kg/m ^2^) or “obesity” (≥ 30 kg/m ^2^). Calculation of the weekly GWG responded to the following quotient: (Weigh at current contact - Weight at previous contact) / Time elapsed between both contacts (in weeks). 

### Data analysis

The descriptive analysis of the characteristics of the sample is presented through mean and standard deviation for the quantitative variables and resorting to number and percentage for the categorical ones. Evolution of the PA (measured in METs/day and in minutes/day) by setting where it is performed throughout pregnancy was tested by means of the T test for related samples. The weekly gestational weight gain comparison by PA level in each of the trimesters of pregnancy was performed by means of the ANOVA test (Bonferroni *post-hoc*). 

The analysis of the factors associated with low PA levels in each of the trimesters of pregnancy was performed by means of different binary logistic regression models (Wald stepwise method with entry probability of 0.05).

Finally, different multiple linear regression models were performed (stepwise method with F-probability for entry of a variable into the model ≤ 0.05) in order to determine the predictive factors of gestational weight gain in each of the trimesters of pregnancy, and analyzing the influence of low PA levels on such gain. A collinearity analysis was performed to remove from each regression model those factors that did not present tolerance and Variance Inflation Factor (VIF) values close to 1 or condition indices below 30. All calculations were performed in the Statistical Package for Social Sciences (SPSS), version 21.0. p < 0.05 was accepted as statistically significant in all the cases.

### Ethical considerations

Prior to launching the study, authorization was requested to the Aragon Ethical Committee of Clinical Research. From a first moment, the participants were informed about the objectives, methodology, potential risks derived from their participation in the study, and their right to withdraw from it at any time. In addition, all the participants signed and informed consent form before initiating their collaboration in the study.

## Results

### Characteristics of the sample

A total of 151 of all 220 women recruited finished the study protocol. The participants’ mean age was 30.77 years old and their pre-gestational BMI was 23.18 kg/m ^2^. By pre-gestational BMI categories, around 70% had normal weight and almost 30% were overweight or obese before their pregnancies. 

Approximately half of the participants were primiparous, lived in rural areas, and had some child under their care. One-fourth of them had already undergone some miscarriage. In addition, most of them were of native origin, had a paid job at the beginning of their pregnancies and did not smoke (Table 1). 

**Table 1:** Characteristics of the sample (n^*^ = 151). Zaragoza, AR, Spain, 2021

Variable	Mean (Standard Deviation)	Number (%)
Age (years old)	30.77 (4.178)	
Pre-gestational Body Mass Index	23.18 (3.130)	
Body Mass Index by categories		
Low weight		3 (2%)
Normal weight		105 (69.5%)
Overweight		33 (21.9%)
Obesity		10 (6.6%)
Parity		
No		80 (53%)
Yes		71 (47%)
History of previous miscarriage		
No		107 (70.9%)
Yes		44 (29.1%)
Area of residence		
Rural		87 (57.6%)
Urban		64 (42.4%)
Geographic origin		
Native		111 (73.5%)
Immigrant		40 (26.5%)
Schooling level		
Elementary School		33 (21.9%)
High School		66 (43.7%)
University studies		52 (34.4%)
Paid job at the beginning of pregnancy		
No		36 (23.8%)
Yes		115 (76.2%)
Smoker		
No		112 (74.1%)
Yes		39 (25.9%)

* n = Number of participants

### PA throughout pregnancy

In our sample, the time devoted to PA gradually decreased throughout pregnancy, as well as its intensity (Table 2). During the 1st and 2nd trimesters of pregnancy, the most time was devoted to moderate PA, whereas in the 3rd trimester walking was the most usual practice. 

**Table 2:** Time (minutes/day) and intensity of the physical activity performed (n^*^ = 151). Zaragoza, AR, Spain, 2021

	1 st trimester (1T) Mean (Standard Deviation)	2 nd trimester (2T) Mean (Standard Deviation)	3 rd trimester (3T) Mean (Standard Deviation)	p-value ^†^
Walking	47.29 (42.1)	39.90 (34.9)	34.65 (22.3)	<0.01 (1T>2T>3T)
Moderate physical activity	56.08 (43.2)	41.82 (30.6)	29.29 (20.3)	<0.01 (1T>2T>3T)
Vigorous physical activity	2.32 (1.96)	1.85 (1.88)	1.84 (1.92)	NS^‡^
Total	105.69 (57.3)	83.57 (42.4)	65.78 (28.0)	<0.01 (1T>2T>3T)

* n = Number of participants;

† p-value = T test for related samples;

‡ NS = Not significant

In all the settings where PA was performed, a significant reduction was observed in the metabolic expenditure associated with PA, except for playful PA which, as pregnancy progressed, showed a higher percentage contribution to the total expenditure derived from the PA performed (Table 3). In any case, the metabolic expenditure derived from the PA performed at home was predominant in each of the trimesters of pregnancy. 

**Table 3:** Evolution of physical activity (measured in METs * /day) throughout pregnancy (n † = 151). Zaragoza, AR, Spain, 2021

	1 st trimester (1T) Mean (Standard Deviation)	2 nd trimester (2T) Mean (Standard Deviation)	3 rd trimester (3T) Mean (Standard Deviation)	p-value ‡
Playful physical activity	28.06 (47.87)	33.96 (34.85)	66.31 (36.35)	<0.01 (3T>2T>1T)
Transportation-related physical activity	57.84 (69.76)	35.72 (39.51)	23.36 (24.80)	<0.01 (1T>2T>3T)
Physical activity at home	128.01 (95.16)	102.32 (68.15)	84.68 (103.23)	<0.01 (1T>2T>3T)
Physical activity at work	126.84 (177.8)	93.41 (142.17)	38.90 (99.95)	<0.01 (1T>2T>3T)
Total physical activity	340.78 (198.31)	265.78 (145.35)	213.01 (101.01)	<0.01 (1T>2T>3T)

* METs = Metabolic Equivalents of Task;

† n = Number of participants;

‡ p-value = T test for related samples

The only factor that was independently related to low PA was the participants’ origin. Thus, foreign origin was inversely associated with low PA levels, with Odds Ratio (95% Confidence Interval) values of 0.209 (0.060 - 0.728), 0.197 (0.065 - 0.594) and 0.232 (0.095 - 0.569) in the 1 ^st^, 2 ^nd^ and 3 ^rd^ trimesters, respectively. Data not available in the tables. 

### Factors related to GWG. Influence exerted by PA

In our sample, weekly weight gain was gradually increased throughout pregnancy, with the highest values in the 3rd trimester. In the bivariate study of the association between PA and GWG only a statistically significant difference was observed. In the 3rd trimester of pregnancy, low PA levels were related to higher weight gains (p < 0.05). Pregnant women with high and average PA levels gained a mean of 169 and 99 grams less per week, respectively (Table 4). 

**Table 4:** Weight gain by level of physical activity performed during pregnancy (n^*^ = 151). Zaragoza, AR, Spain, 2021

	1 st trimester		2 nd trimester		3 rd trimester	
	Number (%)	Kilograms/week (Standard Deviation)	Number (%)	Kilograms/week (Standard Deviation)	Number (%)	Kilograms/week (Standard Deviation)
High level of physical activity	35 (23.1%)	0.206 (0.10)	23 (15.2%)	0.311 (0.13)	8 (5.3%)	0.349 (0.09)
Average level of physical activity	82 (54.3%)	0.190 (0.13)	84 (55.6%)	0.304 (0.11)	83 (54.9%)	0.419 (0.13)
Low level of physical activity	34 (22.5%)	0.180 (0.15)	44 (29.1%)	0.337 (0.11)	60 (39.7%)	0.518 (0.14)^†^
Total	151 (100%)	0.191 (0.13)	151 (100%)	0.315 (0.11)	151 (100%)	0.455 (0.14)

* n = Number of participants;

† In the 3^rd^ trimester, weekly weight gain in the group of women with Low PA was higher than in the Moderate and High PA groups (p<0.05)

In the multivariate study, the factor that was most closely related to GWG was pre-gestational BMI, observing and inverse relationship between both factors in each of the trimesters of pregnancy. In the 1st trimester, the participants with paid jobs outside their homes presenter better GWG. In the 3rd trimester, a higher GWG was observed in the women with some child under their care. In relation to the influence of the PA performed on GWG, the results of the bivariate analysis were confirmed. A significant association between low PA levels and higher weight gains was only observed in the 3rd trimester of pregnancy (Table 5). 

**Table 5:** Multiple linear regression models. Predictors of weekly weight gain by trimesters of pregnancy (n^*^ = 151). Zaragoza, AR, Spain, 2021

Group	Variable	B ^†^ (95% Confidence Interval)	β ^‡^	p-value (Model)
1st trimester	Pre-gestational Body Mass Index§	-0.009 (-0.015, -0.002)	-0.204	0.005
	Paid job outside the house	-0.049 (-0.098, -0.000)	-0.158	
2nd trimester	Pre-gestational Body Mass Index§	-0.059 (-0.101, -0.018)	-0.225	0.005
3rd trimester	Pre-gestational Body Mass Index^§^	-0.013 (-0.020, -0.007)	-0.285	
	Low physical activity	0.105 (0.062, 0.147)	0.351	0.000
	1 or more children under their care	0.064 (0.022, 0.105)	0.219	

* n = Number of participants;

† B = B regression coefficient;

‡ β = Beta coefficient;

§ Pre-gestational Body Mass Index = It is included in the regression model as a continuous quantitative variable

The models’ ability to explain GWG was around 7% (R ^2^= 0.069) for the 1 ^st^ trimester, 5% (R ^2^= 0.051) for the 2nd trimester and 25% (R2 = 0.243) for the 3rd trimester of pregnancy (Table 5). 

## Discussion

The objective of this research was to assess PA levels throughout pregnancy and determine their influence on GWG. In our sample, both the time devoted to PA and its derived metabolic expenditure were significantly reduced as pregnancy progressed. In addition, the only factor independently related to the PA performed was the participants’ origin. On the other hand, pre-gestational BMI was the only factor that exerted a significant influence on GWG at each of the measuring moments, showing an inverse relationship. PA was only related to GWG at the end of pregnancy. Thus, pregnant women with low PA levels presented higher weight gains in the 3 ^rd^ trimester of their pregnancies. 

In previous studies, reductions in the PA levels have been reported throughout pregnancy(25-26). This decrease had been previously attributed to the reduction in the work-related PA levels that can take place in the second half of pregnancy ^(27)^ . In our sample, reductions were evidenced in all the settings where PA was performed, except for playful activities. The environment that most contributed to total metabolic expenditure corresponded to the home, in line with what has already been reported by other authors ^(28)^ . 

In this study, having a paid job outside the house was a factor associated with lower GWG during the 1 ^st^ trimester. As a possible explanation for this finding, the already published literature suggests that unemployment might reflect a population group with more limited economic resources ^(29)^ and lower schooling levels ^(30)^ and, therefore, with less potential for managing their own health, also in relation to pregnancy. 

The study of the effects of the PA performed during pregnancy on GWG has yielded divergent results previously. In previous studies ^(17, 31)^ with a similar methodology to the one used in this research, no significant association was found between both variables. On the other hand, a study conducted in China with 862 pregnant women evidenced lower weight gains in those that were more active during the second and third trimesters of their pregnancies ^(19)^ ; in turn, another survey conducted in Vietnam ^(32)^ indicated lower weight gains only in the pregnant women that were more active during the 3 ^rd^ trimester of their pregnancies. In this same line, in our study only “low” PA levels during the 3 ^rd^ trimester of pregnancy were significantly related to our participants› weight gain. Consequently, the only factor that was independently related to GWG throughout pregnancy was pre- gestational BMI, showing an inverse relationship. This trend has already been reported in previous studies ^(33- 34)^ . A possible explanation for this finding is that women with lower BMI values might feel that pregnancy lowers their responsibility to control their weight, allowing overeating. On the other hand, health educators, obstetric nurses (Midwives) in our environment, are probably more constant in their recommendations regarding GWG in women with higher pre-gestational BMI values. In any case, it is necessary to conduct more research studies in order to understand the biological and/or behavioral origin of this phenomenon. 

The study presents a number of limitations. The PA assessment was performed with a questionnaire based on recall of the activity performed during the last seven days. Consequently, and although IPAQ has already shown a high correlation (r = 0.917) with objective measuring instruments ^(35)^ , certain memory bias cannot be disregarded for this variable. Likewise, using self-reported weight values can induce errors. This is a usual circumstance in this type of studies and inherent to the difficulty determining pre-gestational weight. Nevertheless, high correlations (r > 0.92) have already been observed between measured and reported weight ^(36)^ , as well as actual differences lower than 1 kilogram between both types of measurement ^(37)^ , which justify substituting the measured data with the self-reported ones. Finally, this study does not include some potentially confounding factors such as 1 ^st^ trimester nausea or the result of the glucose tolerance tests, which can under- or over-estimate the influence exerted by PA on GWG. The association between diet and weight evolution is not analyzed either, although it is a determining factor of weight in the general population. Based on these limitations, it seems necessary to conduct new research studies aimed at understanding the determinants of PA and weight evolution during pregnancy and that include an objective measurement of PA and new variables with a potential influence on women’s weight. 

In any case, we honestly believe that this study supposes and advance in scientific knowledge for several reasons. In the first place, it is a pioneering study in Spain in terms of the prospective analysis of the changes in pregnant women’s PA and its influence on GWG. Secondly, the methodology employed represents an improvement over the one used in most of the previous studies that have analyzed PA during pregnancy, which only report global PA numbers or a simple categorization of it. In this sense, the main contributions of this research are two. The first corresponds to the relative conceptualization of the GWG variable in kg/week, a relative and consequently more accurate indicator of weight gain; and the second, to the determination of both a global PA calculation and of the PA performed by settings and by intensity.

We consider that this research carries with it important implications for nurses specialized in Obstetrics (Midwives), who, in our context, are the main health and lifestyle educators for pregnant women. Thus, three important performance lines that imply a training challenge for Nursing professionals are inferred from our results, namely: 1. The need to monitor women’s PA during pregnancy, as well as its predictive factors; 2. Launching initiatives that promote PA during pregnancy; and 3. The convenience of adopting relative GWG monitoring measures (weight gain/time, or individualized recommendations according to criteria set forth by the Institute of Medicine) ^(38)^ to discard the traditional monitoring and counseling practices based on final objectives of weight gains measured in kilograms at the end of pregnancy. In this sense, a recent study ^(39)^ indicates that, although most midwives advise on GWG and PA, their suggestions are often limited to the first contact and are in disagreement with the Institute of Medicine recommendations. 

## Conclusion

The pregnant women in our context have their PA levels reduced throughout pregnancy, except for playful PA, which increases towards the third trimester. The relationship between GWG and the PA performed seems to be limited to the last stages of pregnancy, when less active pregnant women tend to gain more weight.

It is expected that these findings contribute for nurses specialized in Obstetrics (Midwives) to review their beliefs and motivations about the convenience of adequately monitoring GWGW and promoting suitable PA according to the characteristics and evolution of each pregnancy.
